# Secondary cancer risk in head‐and‐neck cancer patients: A comparison of RBE‐weighted proton therapy and photon therapy

**DOI:** 10.1002/mp.17705

**Published:** 2025-02-19

**Authors:** Peter Dasiukevich, Sebastian Tattenberg, Cornelia Hoehr, Abdelkhalek Hammi

**Affiliations:** ^1^ School of Physics and Astronomy The University of Edinburgh Edinburgh UK; ^2^ TRIUMF Vancouver British Columbia Canada; ^3^ School of Natural Sciences Laurentian University Sudbury Ontario Canada; ^4^ Department of Physics Technical University of Dortmund Dortmund Germany

**Keywords:** computational human phantom, Monte Carlo simulation, out‐of‐field dose, proton therapy, secondary cancer

## Abstract

**Background:**

Secondary cancer is a serious side effect from external beam radiotherapy (EBRT). Conventional EBRT is performed using a beam of photons, however, due to their ability to produce more conformal dose distributions, the use of protons is becoming more wide‐spread. Due to this sparing it would be expected that proton therapy could be associated with lower secondary cancer rates compared to photon therapy. However, since proton therapy data is still being accumulated and the follow‐up period is often relatively short thus far, simulation studies can complement the existing data and extrapolate to longer time frames.

**Purpose:**

This study aims to estimate and compare the risk of secondary cancer when treating head‐and‐neck cancer patients with proton therapy or photon therapy, while combining a whole‐body computational human phantom with the patient treatment planning computed tomography (CT) scan in order to study organs that are partially or fully outside of the treatment planning CT. In addition, proton therapy secondary cancer rates are investigated further by including variable relative biological effectiveness (RBE) models.

**Methods:**

For 20 head‐and‐neck cancer patients, two clinical radiotherapy treatment plans were created, one for proton therapy and one for photon therapy. For proton therapy, linear energy transfer (LET) distributions were simulated and used to calculate the variable RBE‐weighted dose distributions for six different variable RBE models, in addition to the constant RBE of 1.1 widely used clinically. In order to obtain the dose deposited outside the treatment planning CT scan, an adjustable whole‐body digital reference phantom was stitched to the treatment planning CT. Based on the resulting dose distributions, the risk of secondary cancer was calculated for each modality.

**Results:**

Averaged across all patients and relevant organs, photon therapy compared to proton therapy with a constant RBE of 1.1 was estimated to be 1.8 times more likely to cause secondary cancer. This risk ratio varied between 1.6 and 2.0, depending on the variable RBE model used. Cases with lifetime attributable risk (LAR) values below 0.1% were excluded from this analysis to prevent the benefits of proton therapy (the ratio $\frac{LAR_{photon}}{LAR_{proton}}$) from being artificially elevated in cases in which $LAR_{proton}\approx 0$.

**Conclusion:**

Proton therapy was associated with lower estimated secondary cancer rates compared to photon therapy when treating head‐and‐neck cancer patients. This trend was observed even when considering different variable RBE models to calculate the proton therapy dose distributions.

## 1 INTRODUCTION

External beam radiotherapy (EBRT) is a standard modality of cancer treatment that aims to destroy the tumor by using a beam of ionization radiation. Although the effects of EBRT are generally very localized, inevitable exposure to the surrounding healthy tissues, peripheral blood, and out‐of‐field doses result in additional cell damage.^1^ This damage to healthy tissue cells increases the chance that a secondary cancer will occur in the patient and therefore, should be reduced as much as possible.^2^ With approximately 1 in 6 people who are diagnosed with cancer having had cancer previously, and with radiotherapy have been identified as a considerable risk factor, secondary cancers are a serious side effect from radiotherapy cancer treatment that needs to be accounted for.^3^, ^4^, ^5^, ^6^, ^7^, ^8^

Proton therapy is a form of EBRT in which the tumor is irradiated with protons, which, due to the Bragg peak, can achieve more conformal dose distributions compared to conventional photon therapy.^9^ These more conformal dose distributions can lead to a lower exposure to the surrounding healthy tissue and therefore reduce the patient's chance of developing secondary cancer. However, since proton therapy data is still being accumulated and the follow‐up period is often relatively short, additional simulation studies are required to support this data and study longer time frames.^10^

A variety of studies have estimated secondary cancer rates for different treatment modalities, including comparisons between photon and proton therapy, with especially pencil‐beam scanning proton therapy having been found to be advantageous compared to conventional photon treatments.^11^, ^12^, ^13^, ^14^, ^15^, ^16^, ^17^, ^18^, ^19^, ^20^, ^21^, ^22^, ^23^, ^24^, ^25^, ^26^, ^27^ However, common limitations of such studies include the use of dose distributions from the treatment planning software determined based on the analytical pencil‐beam algorithm, which may yield less accurate dose distribution compared to Monte Carlo (MC) simulations, especially in heterogeneous regions, as well as being limited to the in‐field volume (IFV), the volume inside the treatment planning computed tomography (CT) scan, without consideration of any contribution to secondary cancer from the out‐of‐field volume (OFV) dose.^28^, ^29^ Alternatively, inclusion of the OFV may be achieved very approximately, for example via use of a computational phantom of a size not matching the patient in question. In addition, proton therapy dose calculations generally rely on the clinically‐used relative biological effectiveness (RBE) value of 1.1 rather than on models which describe the RBE as a function of factors such a dose, cell radiosensitivity, and linear energy transfer (LET).^30^, ^31^

The aforementioned photon and proton therapy comparisons were performed for a variety of different treatment sites, but for head‐and‐neck cancer specifically, Jain et al. compared the estimated risk of developing secondary cancer after photon or proton therapy based on 13 patients with oropharyngeal cancer.^32^ This study found that proton therapy can achieve similar target coverage to intensity modulated radiotherapy (IMRT) while significantly reducing the dose to healthy organs and also reducing the risk of secondary cancer. This work found that, within their patient cohort, four excess cases of secondary cancer per 100 patients could be avoided by treating them with proton therapy instead of IMRT. This work did not calculate dose distributions with MC simulations and did not include the dose deposited in the OFV, which contributes to secondary cancer probabilities.^33^

In the clinic, protons are typically assumed to have a constant RBE of 1.1. However, RBE for protons has been shown to vary throughout the patient.^30^, ^31^ Several phenomenological RBE models have been proposed based on dose, LET, and cell radiosensitivity to predict how RBE varies throughout the patient, offering a more realistic representation of the dose distribution compared to the constant RBE of 1.1 used in clinical settings.

Our work aims to expand upon the above‐mentioned and similar studies in several ways.^11^, ^12^, ^13^, ^14^, ^15^, ^16^, ^17^, ^18^, ^19^, ^20^, ^21^, ^22^, ^23^, ^24^, ^25^, ^26^, ^27^ Firstly, by extending the original treatment planning CT scan with a hybrid computational whole‐body phantom, chosen according to a patient parameters such as sex and size.^13^, ^26^, ^34^ While the resulting hybrid patient CT scan contains the original planning CT, it also allows for the estimation of the out‐of‐field dose distribution to the peripheral organs that are not included in the planning CT. In addition, the dose distributions from the treatment plans are calculated using MC simulations to provide more accurate dose values, and this work will also include further analysis on the proton therapy results, with the inclusion of several variable RBE models.

## 2 METHODS

### 2.1 Dataset

Twenty randomly chosen head‐and‐neck cancer patients were analyzed for this study. These patients are provided in the Cancer Imaging Archive, in the Head‐Neck‐PET‐CT dataset.^35^, ^36^, ^37^ This dataset was chosen as there is a large number of patients (298), head‐and‐neck cancer is a common type of cancer, with head‐and‐neck squamous cell carcinoma accounting for approximately 4.5% of cancer diagnoses as well as cancer deaths, and several important organs are in and near the head‐and‐neck region.^38^ Provided patient data included patient treatment planning CT scans as well as manually delineated radiotherapy targets and organs‐at‐risk (OARs).

### 2.2 Extended full‐body hybrid patient model

**FIGURE 1:**
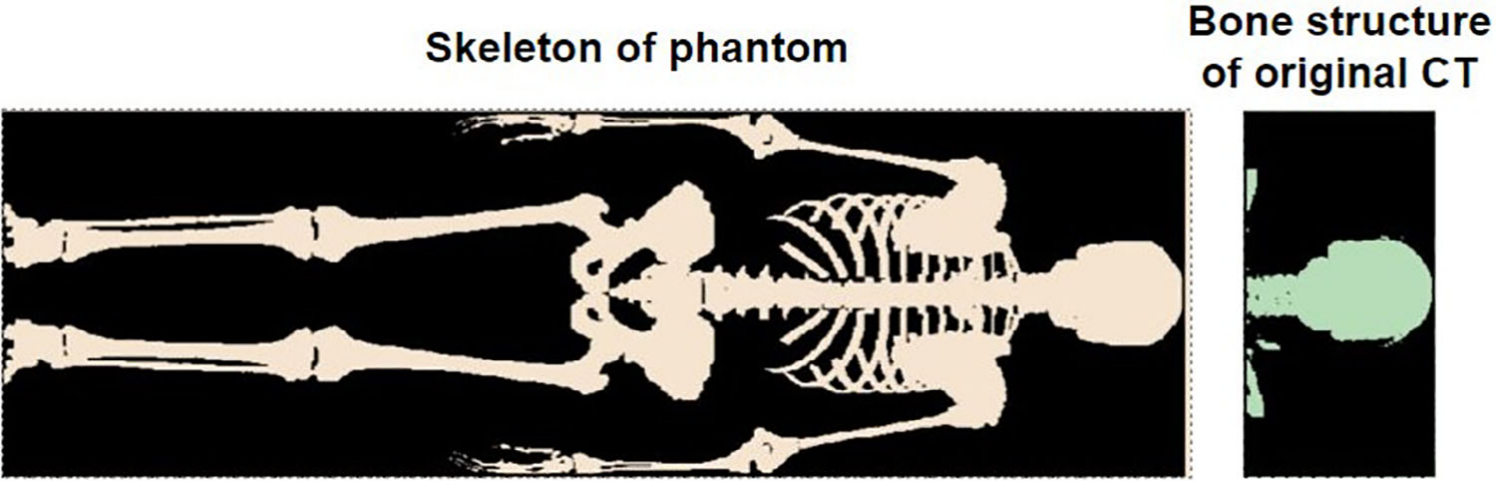
An example of the skeleton of the phantom and the bone structures in the original patient CT that were used to align the original treatment planning CT to the computational phantom. CT, computed tomography.

**FIGURE 2:**
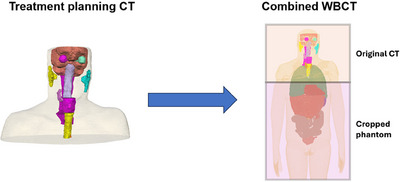
An illustration of the conversion from the treatment planning CT to the extended (WBCT). The various colored contours correspond to a variety of delineated OARs. CT, computed tomography; OARs, organs‐at‐risk; WBCT, whole‐body patient CT.

Since routinely patient CT scans only cover the region including and in proximity to the tumor so that the imaging dose delivered to the patient is minimized, the planning CT alone often does not provide enough anatomical detail to accurately estimate the risk of secondary cancers in peripheral organs. To represent the patient in the OFV, in accordance with the methodology applied by Gallagher et al., the patient's CT scan was supplemented with whole‐body reference computational phantoms provided by the National Cancer Institute.^34^, ^39^, ^40^ This anatomical extension allowed for the creation of a hybrid whole‐body CT (WBCT) specific to the patient, enabling the calculation of secondary cancer rates for organs located in the OFV. The reference phantoms were pre‐selected from the Library of Computational Human Phantoms to match the patient's size and sex. Since no pediatric patients were included in this study, a voxelized phantom representing a 35 year‐old adult was selected. The voxelized geometry of the phantom was mapped to the real‐world coordinate system using the voxel resolution of the selected phantom.^29^, ^39^ To automatically identify the region in the reference phantom that matched the patient anatomy from the planning CT, the reference phantom was then co‐registered to the treatment planning CT based on the overlap of bone structures. Due to the multi‐modality of the data, bone structures and the skeleton were extracted from the planning CT and the phantom data, respectively, and then masked as seen in Figure 1. A simulated annealing approach was implemented to calculate the optimal affine transformation that maximized the structural overlap between the patient's bone structures and the corresponding skeleton in the phantom. This transformation was then applied to match the phantom to the treatment room coordinates based on the planning CT dataset. The registered phantom was subsequently divided along its superior‐inferior axis into two parts: one above and one below the first treatment planning CT slice. The non‐overlapping portion was then fused with the original patient treatment planning CT at this slice to replace the cropped body segment, and a new patient‐specific whole‐body hybrid CT was created preserving the entire spatial information from the original planning CT, as seen in Figure 2. Automatic delineation of organs that were partially in between the IFV and OFV, such as the esophagus and the lungs, so that an estimate for the dose to these organs could be obtained and analyzed, was performed using TotalSegmentator, an open‐source software that uses machine learning to contour various organs on a patient CT scan.^41^ The training dataset used contained CT scans from different scanners, institutions and protocols with 1228 subjects, providing the model with robustness to the variations observed in potential imaging input data.

### 2.3 Treatment planning and dose distribution calculation

**FIGURE 3:**
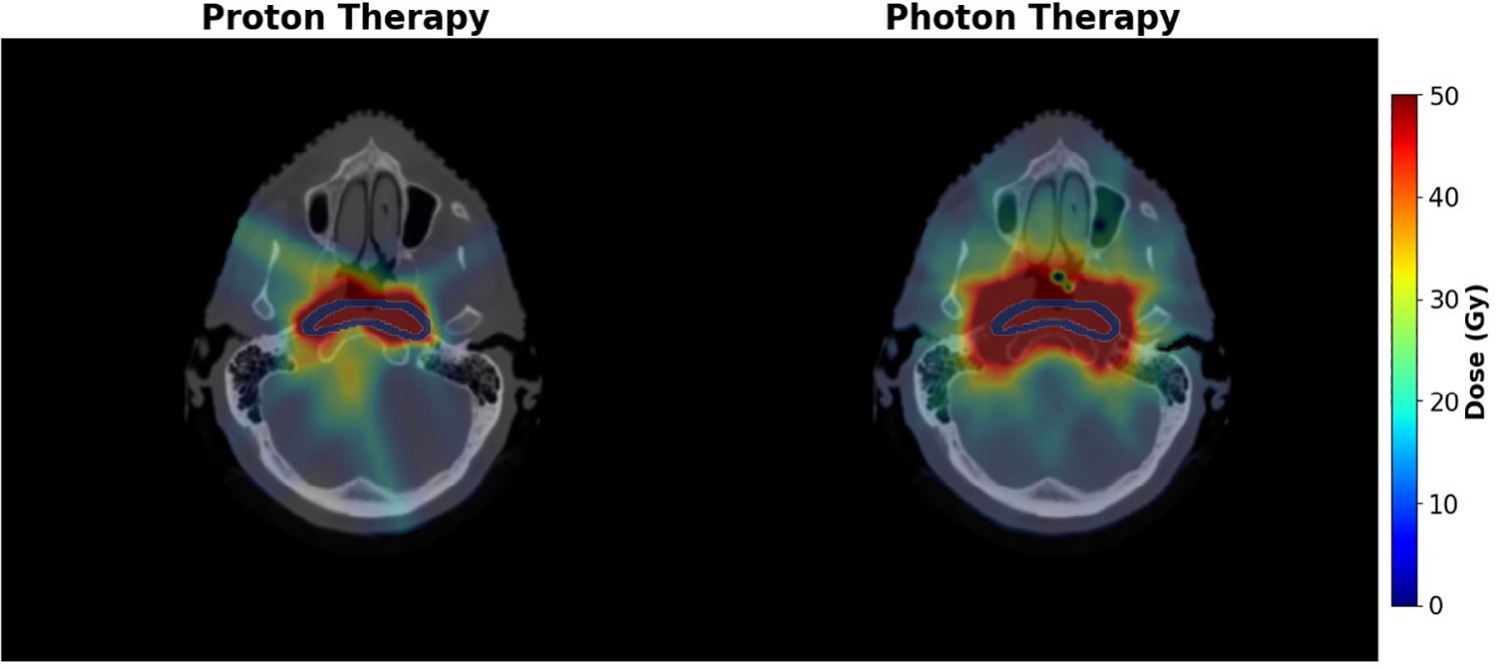
Both dose distributions delivered by the radiotherapy treatment plans for patient 9, depicted in a slice of the IFV. The target contour is shown in blue. A constant RBE value of 1.1 was applied to yield the proton therapy dose distribution. RBE, relative biological effectiveness; IFV, in‐field volume.

For each of the 20 patients, one IMRT and one proton therapy treatment plan was then created in matRad, an open‐source treatment planning system built in Matlab, based on each patient's WBCT.^42^ The clinical objectives and constraints specifying the desired target coverage and OAR sparing were taken from Water et al., and were exactly the same for photon and proton therapy.^43^ For proton therapy, in accordance with clinical practice, the gantry angles used were 60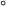, 160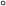, 220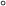, 300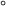  and for photon therapy, a total of 9 gantry angles were used, from 0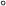 to 360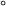  with a 40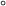 spacing between each one.^44^, ^45^ For photon and proton treatment plan calculations, MC simulations were performed in matRad, with the available extensions for ompMC (an open‐source photon dose calculation engine developed at the Pontifical Catholic University of Chile in Santiago, Chile) and TOPAS (a Geant4‐based toolkit to simulate particle transport and radiation ionization for medical applications), respectively.^42^, ^46^, ^47^ After an initial proton therapy treatment plan optimization run, target objective weights were iteratively modified and the optimization was re‐run to assure comparable target coverage between the photon therapy and the proton therapy treatment plan. For the optimisation of the proton therapy treatment plans, a constant RBE of 1.1 was used. Figure 3 shows the dose distributions produced by the treatment plans for both modalities for patient 9.

### 2.4 Variable RBE models

For proton therapy, in addition to using a constant RBE of 1.1, variable RBE models were used to recalculate the dose distribution based on factors such as the accompanying LET distribution. These models depend on the physical dose deposited in each voxel, the dose‐averaged LET in that voxel and the radiosensitivity parameters for the cell type in question. The dose‐averaged LET ($LET_{d}$) in each voxel was obtained from the aforementioned simulations. Six different variable RBE models were included to allow for a comparison to be made between each model.^48^, ^49^, ^50^, ^51^, ^52^, ^53^ All models used the following equation in their RBE calculation:

(1) $$\begin{array}{ccc} & & RBE\left[D_{p},{\left(\frac{\alpha }{\beta }\right)}_{x},LET_{d}\right]\\ & & =\frac{1}{2D_{p}}\left(\sqrt{{\left(\frac{\alpha }{\beta }\right)}_{x}^{2}+4D_{p}{\left(\frac{\alpha }{\beta }\right)}_{x}RBE_{max}+4RBE_{min}^{2}D_{p}^{2}}-{\left(\frac{\alpha }{\beta }\right)}_{x}\right) \end{array}$$

Where $\alpha_{x}$ and $\beta_{x}$ are cell killing parameters, $\alpha_{x}/\beta_{x}$ was set to 3 for all healthy tissues, and $D_{p}$ is the dose per fraction.^49^ Each model used a different equation defining $RBE_{max}$ and $RBE_{min}$, as shown in Table 1.

**TABLE 1:** Definitions of all variable RBE model used.

Model	$RBE_{max}$	$RBE_{min}$	$p_{0}$	$p_{1}$	$p_{2}$	$p_{3}$
Carabe et al.^48^	$p_{0}+p_{1}\frac{LET_{d}}{{\left(\alpha /\beta \right)}_{x}}$	$p_{2}+p_{3}\frac{LET_{d}}{{\left(\alpha /\beta \right)}_{x}}$	0.843	0.413644	1.09	0.01612
McNamara et al.^49^	$p_{0}+p_{1}\frac{LET_{d}}{{\left(\alpha /\beta \right)}_{x}}$	$p_{2}+p_{3}\sqrt{{\left(\alpha /\beta \right)}_{x}}LET_{d}$	0.99064	0.35605	1.1012	−0.0039
Peeler et al.^50^	$p_{0}+p_{1}\frac{LET_{d}^{3}}{{\left(\alpha /\beta \right)}_{x}}$	$p_{2}+p_{3}{\left(\alpha /\beta \right)}_{x}LET_{d}^{3}$	0.75	0.00143	1.24	0.00074
Tilly et al.^51^	$1+p_{0}\frac{LET_{d}}{{\left(\alpha /\beta \right)}_{x}}$	1.0	0.309 or 0.550964	—	—	—
Rørvik et al.^52^	$p_{0}+p_{1}\frac{LET_{d}}{{\left(\alpha /\beta \right)}_{x}}$	1.0	1.0	0.645	—	—
Wedenberg et al.^53^	$p_{0}+p_{1}\frac{LET_{d}}{{\left(\alpha /\beta \right)}_{x}}$	1.0	1.0	0.434	—	—

*Note*: For Tilly et al., two $p_{0}$ values were included, one for low and one for high ${\left(\alpha /\beta \right)}_{x}$, of which our work used the former.

Abbreviation: RBE, relative biological effectiveness.

For proton therapy, seven different RBE‐weighted dose distributions were calculated, which were then used to estimate seven corresponding secondary cancer rates (one of which assumed a constant RBE of 1.1), allowing for an analysis to be performed on the sensitivity of calculated secondary cancer rates with respect to the RBE model used.

### 2.5 Calculation of secondary cancer risk

Once the dose distributions in the patients were calculated, the risk of radiation‐induced secondary cancer was calculated based on the formalism of organ equivalent dose proposed by Schneider et al.^54^, ^55^ This was done by first calculating the organ equivalent dose (OED) for each organ. Two models for calculating the OED were used, one for sarcoma and one for carcinoma. Carcinomas typically form in epithelial cells that line organs while sarcomas form in connective tissue and bone.^56^ The carcinoma and sarcoma OED values were calculated with Equations 2 and 3, respectively.

(2) OEDc=1V∑iVie−αi′Diαi′R1−2R+R2eαi′Di(1−R)2e−αi′R1−RDi−(1−R)2e−αi′R1−RDi

(3) OEDs=1V∑iVie−αi′Diαi′R1−2R+R2eαi′Di−αi′RDi(1−R)2e−αi′R1−RDi−(1−R)2e−αi′R1−RDi

In Equations 2 and 3, i is the voxel number, V is the total organ volume, $V_{i}$ and $D_{i}$ are volume and dose in each segment, R is the repopulation factor and $\alpha^{\prime }$ is the cell kill parameter which is defined by:

(4) $$\alpha^{\prime }=\alpha +\beta D_{i}\frac{d_{f}}{D}$$

Here, D is the total dose and $d_{f}$ is the dose per fraction. The excess attributable risk (EAR) of developing secondary cancer at a given age was then calculated with the following equation:

(5) $$EAR\left(D,e,a,s\right)=OED.\beta .e^{\left(\gamma_{e}\left[e-30\right]+\gamma_{a}ln\left[a/75\right]\right)}\left(1\pm s\right)$$

In Equation 5, e is the age at which the patient received EBRT, a is the patient's current age, s is a sex factor for male and female, β is the slope of the dose‐response curve in the low‐dose region, and depending on the calculation, OED refers to the carcinoma or sarcoma value calculated according to Equation 2 or Equation 3. Analysis included all potentially relevant organs which had been manually delineated in all patients or for which automatic delineation was possible with the chosen workflow, which was true for the brain, mouth, esophagus, lungs and larynx. Organ parameters were taken from Schneider et al.; for the larynx, organ parameters for the mouth and pharynx were used and for the esophagus, parameter values were taken from Santos et al.^57^, ^58^ Finally, using the EAR, the lifetime attributable risk (LAR) was calculated, which is the percentage chance a patient will develop secondary cancer in their lifetime due to the treatment they received.

(6) $$LAR\left(D,e,a\right)=\int_{a=e+L}^{75}EAR\left(D,e,a,s\right).\frac{S(a)}{S(e)}da$$

Here, $\frac{S(a)}{S(e)}$ is the probability of the patient being alive at the integrating age a if they received cancer treatment at age e, based on United States (US) Life Tables of the general US population.^24^ For each patient, carcinoma and sarcoma LAR values were computed for all photon therapy treatment plans and all proton therapy RBE scenarios. The impact of the different treatment modalities was then assessed and compared.

## 3 RESULTS

**FIGURE 4:**
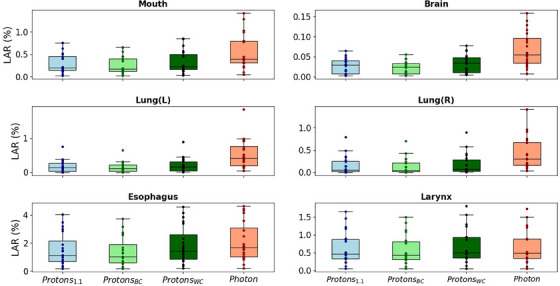
Boxplots of the LAR values for the six OARs considered in this study. Data is shown for proton therapy using a constant RBE of 1.1 ($Proton_{1.1}$), the best‐case variable RBE model ($Proton_{BC}$), the worst‐case variable RBE model ($Proton_{WC}$), and photon therapy (Photon). The line represents the median value across the 20 patients and each datapoint corresponds to an individual patient's LAR value. RBE, relative biological effectiveness; OARs, organs at risk; LAR, lifetime attributable risk.

**FIGURE 5:**
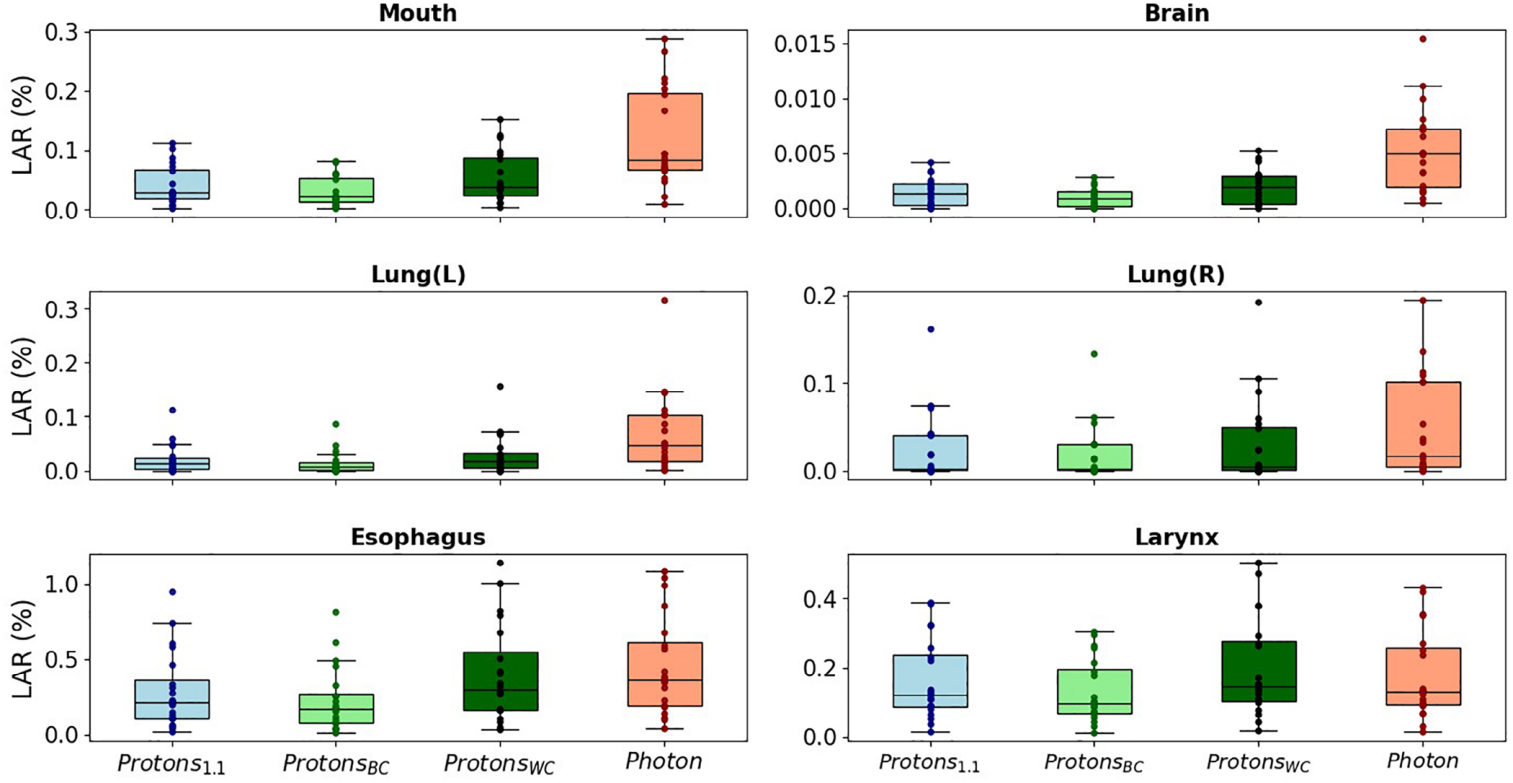
Boxplots of the LAR values for the six OARs considered in this study. Data is shown for proton therapy using a constant RBE of 1.1 ($Proton_{1.1}$), the best‐case variable RBE model ($Proton_{BC}$), the worst‐case variable RBE model ($Proton_{WC}$), and photon therapy (Photon). RBE, relative biological effectiveness; OARs, organs at risk; LAR, lifetime attributable risk.

Figure 4 shows the calculated carcinoma LAR values for all 20 patients, with the worst‐case variable RBE model being the model with the highest LAR values and the best‐case model being the model with the lowest LAR values in any given case. The worst‐ and best‐case variable RBE models those suggested by Rørvik et al. and Peeler et al., respectively, as can be seen in Table 1. The calculated sarcoma LAR values for all 20 patients and evaluated organs can be seen in Figure 5. As reflected in Figures 4 and 5, proton therapy was generally associated with lower LAR values compared to photon therapy. However, this benefit varied considerably depending on the RBE model used. Specifically in the case of the larynx, photon therapy performed better than the worst‐case variable RBE model. Table 2 summarizes the carcinoma results averaged over all patients.

**TABLE 2:** The mean LAR values over all patients and their standard deviations for proton therapy using a constant RBE of 1.1 ($Proton_{1.1}$), the best‐case variable RBE model ($Proton_{BC}$), the worst‐case variable RBE model ($Proton_{WC}$), and photon therapy (Photons).

Modality	Mouth	Larynx	Brain	Esophagus	Lung (L)	Lung (R)
Photons	0.55 ± 0.38	0.64 ± 0.46	0.07 ± 0.04	2.10 ± 1.38	0.54 ± 0.42	0.43 ± 0.35
$Protons_{1.1}$	0.30 ± 0.22	0.63 ± 0.44	0.03 ± 0.02	1.55 ± 1.13	0.18 ± 0.18	0.16 ± 0.20
$Protons_{WC}$	0.33 ± 0.25	0.67 ± 0.48	0.03 ± 0.02	1.86 ± 1.30	0.22 ± 0.21	0.18 ± 0.23
$Protons_{BC}$	0.26 ± 0.19	0.58 ± 0.41	0.02 ± 0.02	1.39 ± 1.02	0.15 ± 0.15	0.14 ± 0.18

Abbreviations: RBE, relative biological effectiveness; LAR, lifetime attributable risk.

When calculating the LAR ratio of the two modalities (the ratio $\frac{LAR_{photon}}{LAR_{proton}}$), a cutoff value of 0.1% for $LAR_{proton}$ was applied to avoid artificially high ratios. This was particularly relevant for organs where $LAR_{proton}$ approached zero, due to the intrinsic dose distribution of protons at especially the distal beam edge. Using the carcinoma results, averaged across all patients and relevant organs, photon therapy compared to proton therapy with a constant RBE of 1.1 was 1.8 (σ = 0.77) times more likely to cause secondary cancer. This benefit was reduced to 1.6 (σ = 0.65) when photon‐based treatment plans were compared to the worst‐case variable RBE model and elevated to 2.0 (σ = 0.89) when compared to the best‐case variable RBE model. Table 3 summarizes the sarcoma results averaged over all patients.

**TABLE 3:** The mean LAR values over all patients and their standard deviations for proton therapy using a constant RBE of 1.1 ($Proton_{1.1}$), the best‐case variable RBE model ($Proton_{BC}$), the worst‐case variable RBE model ($Proton_{WC}$), and photon therapy (Photons).

Modality	Mouth	Larynx	Brain	Esophagus	Lung (L)	Lung (R)
Photons	0.12 ± 0.08	0.18 ± 0.12	0.01	0.451 ± 0.32	0.07 ± 0.07	0.05 ± 0.06
$Protons_{1.1}$	0.04 ± 0.03	0.16 ± 0.11	0.00	0.29 ± 0.25	0.02 ± 0.03	0.02 ± 0.04
$Protons_{WC}$	0.05 ± 0.04	0.20 ± 0.14	0.00	0.40 ± 0.32	0.03 ± 0.04	0.03 ± 0.05
$Protons_{BC}$	0.03 ± 0.02	0.13 ± 0.09	0.00	0.23 ± 0.21	0.02 ± 0.02	0.02 ± 0.03

Abbreviations: RBE, relative biological effectiveness; LAR, lifetime attributable risk.

When analyzing the sarcoma results, averaged across all patients and relevant organs, photon therapy compared to proton therapy with a constant RBE of 1.1 was 1.5 (σ = 0.50) times more likely to cause secondary cancer. This number was increased to 1.7 (σ = 0.49) when applying the the best‐case variable RBE model, but was reduced to 1.1 (σ = 0.33) when considering the worst‐case variable RBE model.

## 4 DISCUSSION

Due to the limited number of available reference phantoms and the inter‐subject variation in anatomy among patients of the same sex and age, the computational human phantoms used in this study did not perfectly match each patient's individual anatomy. However, they provided a reasonable approximation of the anatomical representation of the patient cohort. These reference phantoms, developed by the National Cancer Institute (NCI), are based on medical imaging data from real subjects with body morphology (height and weight) similar to the reference data.^59^ Furthermore, the NCI phantom library includes phantoms that cover a wide range of demographic parameters, such as age, sex, height, and weight, allowing for the selection of the best‐fit phantom for each patient to enhance the accuracy of the patient‐specific hybrid WBCT model, and therefore ensuring reliable out‐of‐field dose estimations.^39^, ^60^

A variety of different secondary cancer models have been suggested, including in the article in which the secondary cancer model used was first presented.^58^, ^61^, ^62^ The model utilized in this work was the full model suggested by Schneider et al., as they found it to provide the best fit for all organs other than the colon, cervix, and skin.^58^ In contrast to some other models, the full model includes the effects of treatment fractionation and thereby considers the impact of tissue repair. One shortcoming of the utilized secondary cancer model with respect to the head‐and‐neck cancer cohort utilized in this study is that the dose delivered to the mouth and pharynx in the patients on which the model was based was only up to 45 Gy, making its use problematic in cases in which the aforementioned dose is exceeded. In addition, as suggested in the original article in which the secondary cancer model utilized was presented, the dose‐response relationship for the whole body was not able to be applied to the patients included in this study, as such uses should be limited to Hodgkin lymphoma patients similar to the ones based on whom the model was originally developed.^58^ The reported genetic predisposition of Hodgkin lymphoma patients towards developing secondary cancers is also of concern.^63^

For the larynx, proton therapy reduced the secondary cancer rates marginally compared to photon therapy. This may be due to the larynx being in close proximity to the tumor and the lower number of beam angles used for proton therapy, since increasing this number provides the optimizer with more degrees of freedom, permitting different trajectories to the target and potentially allowing proton therapy to better spare the larynx.^64^ The largest difference between modalities was observed in the lungs and brain. This may be due to positioning of these organs relative to the tumor, as due to the sharper dose fall‐off in proton therapy, these organs received considerably different dose distributions than during photon therapy. Less of a difference was seen in the esophagus, which may be due to the organ extending closer to the tumor compared to the lungs and brain, wherefore it cannot be spared as much without compromising tumor coverage. Proton therapy was also associated with significantly lower secondary cancer rates in the mouth, however, it should be noted that the mouth was not taken into account during treatment planning and its inclusion may lead to lower secondary cancer rates for both modalities. The worst‐case and best‐case variable RBE models were the models proposed by Rørvik et al. and Peeler et al., respectively. This was due to these two models producing the highest and lowest RBE‐weighted dose distributions, which is in agreement with previous work done by Rørvik et al.^52^

Jain et al. calculated the risk of developing secondary cancer when treating oropharyngeal cancer with proton therapy and photon therapy.^32^ Our study and their work followed a similar methodology, but there were some differences. Their study used a different equation for the calculation of the OED, they did not use MC simulations to obtain dose distributions, and they did not include any dose contributions in the OFV. Despite this, comparing the results from the carcinoma model with their work, there is very good agreement between the results of both works. In the esophagus, they found that photon therapy was 1.48 (σ = 0.08) times more likely to cause secondary cancer, while our results produced a value of 1.45 (σ = 0.34). In the lungs, they had a relative risk of 12.8 (σ = 5.16), while our results showed a relative risk of 5.95 (σ = 5.89). Finally, the only other organ analyzed in both studies was the larynx, for which they found a relative risk of 1.18 (σ = 0.03) while our results produced a value of 1.03 (σ = 0.04). This difference in larynx values may be explained by the gantry angles used, as they used a gantry angle coming from the posterior of the patient, allowing more of the larynx to be spared during proton therapy. The relative importance of planning constraints and objectives placed on the larynx and other structures during radiotherapy treatment planning may also have contributed. No variable RBE models were included in their study, so no comparison can be made with our results in that regard.

## 5 CONCLUSIONS

By using MC simulations to calculate the dose distributions and a WBCT as a hybrid phantom extending the patient treatment planning CT scan into the OFV, the risk of secondary cancer for twenty head‐and‐neck cancer patients was estimated for proton therapy and photon therapy. When averaged over all patients and relevant organs, photon therapy was associated with 1.8 times higher estimated secondary cancer rates compared to proton therapy. A similar trend was still observed when including variable RBE models to calculate the proton therapy dose distribution.

Future works may look to expand upon this study in several ways. Further treatment modalities such as volumetric arc therapy and proton arc therapy could be included, as these treatment modalities have shown to reduce the dose deposited in OARs during treatment.^64^, ^65^ Secondary cancer rates could be calculated for other cancer treatment sites as only head‐and‐neck cancers were included in this work. Pediatric patients could also be included in the patient cohort, as this will increase the period of time for which secondary cancers are being calculated and proton therapy is commonly used for pediatric patients because of the lower secondary cancer rates and better organ sparing associated with the modality.^66^

## CONFLICT OF INTEREST STATEMENT

The authors declare no conflicts of interest.

## Data Availability

The data that support the findings of this study are available from the authors upon reasonable request.
